# Right heart dysfunction does not increase mortality associated with cemented hemiarthroplasty for hip fractures: A retrospective, preliminary analysis

**DOI:** 10.1371/journal.pone.0318993

**Published:** 2025-02-26

**Authors:** Atticus Coscia, Avnee Kumar, Michal Jandzinski, Lauren Swany, Francisco Fuster, Jaimo Ahn

**Affiliations:** 1 Department of Orthopaedic Surgery, University of Michigan, Ann Arbor, Michigan, United States of America; 2 Division of Pulmonary and Critical Care and Sleep Medicine, University of California San Diego, San Diego, California, United States of America; 3 Department of Orthopaedic Surgery, University of Miami, Miami, Florida, United States of America; 4 Department of Orthopaedics, Emory University and Grady Memorial Hospital, Atlanta, Georgia; Carol Davila University of Medicine and Pharmacy: Universitatea de Medicina si Farmacie Carol Davila din Bucuresti, ROMANIA

## Abstract

**Background:**

Cemented hemiarthroplasty (CH) is the preferred treatment for geriatric intracapsular hip fracture. Historically, there has been concern regarding cardiopulmonary morbidity related to bone cement impaction syndrome (BCIS). Right ventricular dysfunction predisposes to BCIS, though limited data supports this association. The purpose of this study was to evaluate if right heart dysfunction was predictive of mortality and cardiopulmonary morbidity following cemented hip hemiarthroplasty.

**Methods and findings:**

This study was a retrospective chart review and analysis conducted at a tertiary referral medical center. Patients who underwent hemiarthroplasty (cemented or press fit) at a single institution for the treatment of intracapsular hip fracture between January 1, 2013, and January 1, 2021 and had an echocardiogram completed within 90 days of their surgery were retrospectively included. Two groups of patients with right heart dysfunction were evaluated: 1) patients who carried a preexisting diagnosis of pulmonary hypertension prior to hospital admission for hip hemiarthroplasty and, 2) patients with echocardiographic evidence of pulmonary hypertension.

The primary outcomes included intraoperative hemodynamic instability consistent with bone cement impaction syndrome (BCIS), in-hospital mortality, and 1-year mortality. N = 74 patients were included. 45 patients underwent cemented hemiarthroplasty and 29 patients received press-fit stems. Preexisting diagnosis of right heart dysfunction and echocardiographic evidence of right heart dysfunction were not significantly associated with BCIS, in-hospital, nor 1-year mortality. However, cementation was significantly associated with 1-year mortality (*p* < 0.05).

**Conclusions:**

Right heart dysfunction does not appear to predispose patients to cardiopulmonary complications or early mortality following cemented hip hemiarthroplasty. Cementation was, however, independently associated with 1-year post-operative mortality. These data add to a growing body of literature which suggests that patients with multiple medical comorbidities may be at risk of adverse outcomes related to cementation. BCIS is uncommon, remains poorly understood, and further investigation is warranted.

## Introduction

Cemented hemiarthroplasty (CH) is the preferred treatment for geriatric patients undergoing surgical management of a displaced intracapsular hip fracture [1,2]. Historically, despite demonstrated benefits in the literature, there has been concern regarding cardiopulmonary morbidity associated with cementation, related to the phenomenon of bone cement impaction syndrome (BCIS) [3–6]. As modern cementation techniques and anesthesia care have evolved, a growing body of literature has shown BCIS to be largely preventable [2,6–9]. However, CH in patients with clinically significant cardiopulmonary disease (such as pulmonary hypertension) remains controversial and several studies have demonstrated higher morbidity and mortality associated with cementation in such patients [3,10–13].

The pathophysiology of BCIS is hypothesized to involve lung embolization of medullary fat and cement resulting in hypoxia and pulmonary vasoconstriction, thereby increasing right ventricular afterload [4,5,14]. Patients with preexisting pulmonary hypertension (PH) are considered to be especially susceptible [4]. Previous work has demonstrated a significant correlation between elevated preoperative pulmonary arterial pressure (>30 mm Hg), as measured by transthoracic echocardiogram (TTE), and the development of BCIS during CH undertaken with outdated cementation techniques [15]. However, to our knowledge, no subsequent study has investigated if preoperative TTE parameters of PH are predictive of BCIS when modern cementation techniques are utilized.

The purpose of this study was to determine if patients with a preexisting diagnosis of PH or evidence of PH on preoperative TTE are more likely to develop BCIS, postoperative cardiopulmonary complications, or mortality after CH. Anecdotally, we have not appreciated adverse outcomes when well-resuscitated patients with PH undergo CH and we thus hypothesized that there would not be an association between PH and adverse intra- or postoperative outcomes, when controlling for other medical comorbidities.

## Methods

This study was structured in accordance with the STROBE statement [16]. This study received institutional review board approval (University of Michigan Medical School Institutional Review Board, HUM00213389) as exempt and the requirement for obtaining informed consent was waived and informed consent was not taken from study participants. A retrospective chart review and analysis was conducted of a tertiary care academic institution’s electronic medical record system. Current Procedural Terminology codes 27125, 27236 and 93306 were used to identify patients who had undergone hip hemiarthroplasty and received a TTE within the hospital system in the period from January 1, 2013, to January 1, 2021. Patients who had a TTE completed within 90 days of undergoing hip hemiarthroplasty for an isolated intracapsular hip fracture (AO/OTA 31-B) were considered for inclusion. Exclusion criteria were incomplete or inadequate information in the patient’s medical record, pathologic fracture, polytraumatized patients, and revision operation.

Data was extracted through electronic medical record review from 2/1/23 through 3/1/23. Data was fully anonymized and no information which could identify individual patients was recorded. Patient’s pre- and post-operative radiographs were reviewed to confirm the diagnosis of intracapsular hip fracture and treatment via hemiarthroplasty. Formal imaging reads by attending radiologists and operative notes were reviewed to ensure that there was no concern for pathologic fracture and to determine the type of stem fixation used (CH versus press fit). The date of the patient’s most recent TTE was reviewed to determine if the study had been completed within 90 days of their surgery. Patients who met inclusion criteria then underwent full chart review.

Patients were grouped according to two variables of interest: cemented versus press fit hemiarthroplasty stem fixation and presence of pulmonary hypertension or right heart dysfunction. Regarding cemented versus press fit hemiarthroplasty stem fixation, the decision to proceed with cemented versus uncemented implants depended entirely on the discretion of the operating surgeon. However, as the evidence supporting cementation has grown, cemented implants have increasingly become the default implant within the orthopaedic department where this study was conducted. Generally, only patients with significant comorbidities or intraoperative hemodynamic instability have received uncemented implants. Fourth-generation cementation techniques, including intramedullary restrictor use, bone bed preparation (pulsatile lavage, brushing, drying), retrograde cement insertion, cement pressurization, cement centrifugation and vacuum mixing, distal stem centralization are standard within the orthopaedic department where this study was conducted [17].

Regarding pulmonary hypertension, two groups of patients with right heart dysfunction were evaluated: 1) patients who carried a preexisting diagnosis of pulmonary hypertension prior to hospital admission for hip hemiarthroplasty and, 2) patients with echocardiographic evidence of pulmonary hypertension. Patients were classified as having pulmonary hypertension if they carried a pre-existing diagnosis prior to admission for surgical management of intracapsular hip fracture. Patients were classified as having echocardiographic evidence of pulmonary hypertension based upon review of TTE reports. TTE reports were reviewed and the patient’s right ventricular systolic pressure and the reading cardiologist’s conclusion regarding the presence of pulmonary hypertension was recorded. Although the gold standard for diagnosis of PH requires right heart catheterization, these echocardiographic parameters are commonly included in standard TTEs and are general indicators of right heart function [18]. Ejection fraction was also recorded. This variable was selected as a general indicator of left heart function and as an indirect indicator of possible type 2 pulmonary hypertension [19]. Patients’ left ventricular ejection fractions (EF) were also classified according to the universal definition of heart failure: heart failure with reduced ejection fraction (EF ≤ 40%), heart failure with mildly reduced ejection fraction (EF 41 to 49%), and heart failure with preserved ejection fraction (EF ≥ 50%) [20].

Data detailing patients’ preexisting comorbidities, medications, and preoperative laboratory markers were recorded. Recorded variables included age, sex, BMI, preadmission residence (assisted versus independent living status), functional status (independent ambulator versus reduced mobility), ASA score, type of anesthesia (regional versus general), presence of medical comorbidities (diabetes, stroke, hypertension, coronary artery disease, left heart failure, aortic stenosis, cancer history, COPD, need for oxygen at home, dementia). Admission hemoglobin (Hgb) and creatinine (Cr) as well as duration from admission to surgery were also recorded. The included variables were selected based on previously described demographic factors and comorbidities associated with postoperative mortality or associated with the development of BCIS [13,21]. The year the patient underwent surgery was also recorded given the time span over which the review was conducted. The primary outcomes recorded via chart review included in-hospital and 1-year mortality. The secondary outcome measure recorded from chart review was development of BCIS. Development of BCIS was determined through review of anesthesia records, which were reviewed for oxygen saturation (measured by pulse oximeter) and systolic blood pressure, which were recorded every 15 minutes. The timing of the cementing process was unreliably recorded in the anesthesia charts. We thus elected to record the lowest systolic blood pressure noted during the final third of the case. The final one third of the case was selected as this period commonly coincides with the portions of the case when BCIS occurs (i.e., cementation, prosthesis insertion, and joint reduction) [4]. The final one third of the case was calculated by dividing the total surgical time (from incision to completion of incisional closure) by three. The lowest systolic blood pressure recorded during the final one third of the case was compared with the first systolic blood pressure recorded following induction and positioning and prior to incision. Each patient was classified as no sequelae of BCIS (Grade 0) or Grade 1, 2, or 3 BCIS according to the classification of Donaldson et al.: Grade 1: moderate hypoxia or hypotension defined as *S*p_O2_ < 94% or fall in SBP > 20%, Grade 2: severe hypoxia or hypotension defined as *S*p_O2_ < 88% or fall in SBP > 40%, Grade 3: cardiovascular collapse requiring cardiopulmonary resuscitation [4].

### Statistical analysis

Data were prescreened to check for outliers and missing data. Assumptions for multiple regression (i.e., linearity, homoscedasticity, independence) were evaluated. Creatine and Total Duration of Stay were nonnormal; therefore, these variables were Winsorized at three standard deviations above the mean for analysis. Descriptive analyses and correlations were conducted for all study variables. Multivariate logistic regression was conducted using the PROC LOGISTIC statement in SAS 9.4 to test study hypotheses (SAS Institute, Inc., Cary, NC, Copyright © 2016). The SAS code utilized for this project has been uploaded as supplementary materials for the purpose of transparency of reporting. Separate models were estimated to test associations between echocardiographic parameters of pulmonary hypertension, preexisting diagnosis of pulmonary hypertension, and cementation, and each dependent variable: 1 year mortality (primary outcome) and BCIS (secondary outcome), for a total of three models. Age, sex, and BMI were included as demographic covariates in all models. Interaction terms for echocardiographic diagnosis of pulmonary hypertension x cementation and preexisting diagnosis of pulmonary hypertension x cementation were entered sequentially. Significant interactions were probed with simple slopes [22].

With respect to additional covariates, correlation coefficients were calculated to determine whether any of the medical comorbidity or echocardiographic covariates collected were significantly related to outcomes of interest. Variables that were not significantly correlated with outcomes were not included in the multivariable logistic regression analysis in concordance with the principle of parsimony. A past medical history of stroke and dementia were each significantly correlated with 1 year mortality and were therefore included as covariates in the multivariate logistic regression models predicting 1 year mortality (primary outcome) None of the past medical history, preoperative laboratory data or ejection fraction data were significantly correlated with BCS I (secondary outcome).

## Results

In total, 549 patients were identified and 74 met inclusion criteria. 471 patients were excluded as they had not had an echocardiogram completed within 90 days of undergoing surgery. 2 patients were excluded due to the presence of pathologic fracture. 1 patient was excluded due to incomplete information included within their chart. A final patient was excluded as they received a revision hemiarthroplasty for performed in the setting of periprosthetic fracture.

### Descriptive statistics

Descriptive statistics are presented in Table 1. Participants ranged in age from 60 to 95 years old. With respect to zero-order correlations between cementation and study variables, cementation was correlated with oxygen dependence at home (r = −0.02, *p* < .05), and year of surgery (r = 0.25, *p* < .05) such that patients who received cementation were more likely to receive oxygen at home and more likely to have undergone surgery later in the study period.(see Table 1). Regarding outcomes, patients who received cemented implants had higher likelihood of 1 year mortality, the study’s primary outcome (*r* = 0.26, *p* < .05; Table 1). There were 4 cases of in-hospital mortality, each case occurring in the setting of cemented implants (Table 2). No in-hospital mortalities occurred in patients who received uncemented implants.

**Table 1:** Descriptive Statistics for main study variables.

Variable	Cemented (N = 45)		Press fit (N = 29)		Correlation
	Mean (N)	SD	Mean	SD	
*Demographics*					
Gender	0.51 (23)	0.51	0.55 (16)	0.51	−0.04
Age	80.58	10.37	78.41	11.47	0.10
Year of surgery (median)	2017	2.84	2015	3.22	0.25*
*Past Medical History*					
ASA	3.36	0.61	3.34	0.70	0.01
BMI	23.95	4.17	25.11	6.13	−0.11
Days from TTE to OR	16.60	26.93	23.66	31.28	−0.12
Assisted living	0.36 (16)	0.48	0.46 (13)	0.51	−0.11
Reduced mobility	0.78 (35)	0.42	0.76 (22)	0.44	0.02
Diabetes Mellitus	0.29 (13)	0.46	0.38 (11)	0.49	−0.09
Stroke	0.32 (14)	0.47	0.31 (9)	0.47	0.01**
Hypertension	0.66 (29)	0.48	0.79 (23)	0.41	−0.14
Coronary artery disease	0.47 (21)	0.50	0.55 (16)	0.51	−0.08
Left heart failure	0.42 (19)	0.50	0.55 (14)	0.63	−0.11
Pulmonary hypertension	0.13 (6)	0.34	0.10 (3)	0.31	0.02
Aortic stenosis	0.04 (2)	0.2	0.17 (5)	0.38	−0.21
Cancer history	0.27 (12)	0.45	0.17 (5)	0.38	0.11
COPD	0.16 (7)	0.37	0.21 (6)	0.41	−0.07
Oxygen at home	0.09 (4)	0.29	0.10 (3)	0.31	−0.02*
Dementia	0.38 (17)	0.49	0.14 (4)	0.35	0.26**
*Admission data*					
Hemoglobin	11.95	2.16	12.63	1.84	−0.16
Creatinine	1.19	0.83	1.37	0.86	−0.10
Delay to OR (days)	1.75	1.31	1.69	1.51	0.02
*TTE Values*					
RV systolic pressure	46.71	11.79	41.55	12.31	0.21
Pulmonary hypertension	0.36 (16)	0.48	0.21 (6)	0.41	0.16
Ejection Fraction	54.21 (42)	14.81	52.82 (28)	19.14	0.04
≥ 50%	(30)		(20)		
49–41%	(0)		(1)		
≤ 40%	(12)		(1)		
*Outcomes*					
BCIS I	0.49 (22)	0.51	0.28 (8)	0.45	0.21
BCIS II	0.04 (2)	0.21	0.03 (1)	0.19	0.02
BCIS III	–	–	–	–	
In-hospital mortality	0.09 (4)	0.29	0	–	0.19
1-year mortality	0.42 (19)	0.50	0.17 (5)	0.38	0.26*

*Note:* Bolded values = * = *p* < 0.05 correlation with cementation status, ** = *p* < 0.05 correlation with 1 year mortality.

**Table 2:** Details of the 4 cases of perioperative death in cemented hemiarthroplasty group.

ASA	Past Medical History	PH on echo	Time to surgery (days)	Time from OR to death (days)	Cause of death
4	COPD (on 3L home O_2_), HTN, CAD	N	3	5	Hypoxemic respiratory failure, aspiration pneumonia
3	CKD3, CAD	N	2	7	Hypercarbic respiratory failure
4	Afib, CHF, COPD (on 3L home O_2_), PH	N	1	5	Status epilepticus, hypoxemic respiratory failure
3	PH (on 2L home O_2)_, CAD, CHF, hx CVA	N	0	7	Hypercarbic respiratory failure, aspiration due to epitaxis

*Note:* CAD: coronary artery disease, CHF: congestive heart failure, CKD: chronic kidney disease, COPD: chronic obstructive pulmonary disease, CVA: cerebrovascular accident, HTN: hypertension, PH: pulmonary hypertension.

### Multivariate logistic regression

Results from multivariate logistic regression are reported in Tables 3 and 4.

**Table 3:** Multivariate logistic regression results (Pulmonary hypertension on echocardiogram).

*Variable*	BCIS 1			1-year mortality		
	*β*	S.E.	Wald Chi-Square	*β*	S.E.	Wald Chi-Square
Intercept	−4.47	2.89	2.39	2.14	3.28	0.43
Gender	0.31	0.50	0.38	−0.25	0.58	0.18
Age	0.02	0.03	0.65	−0.02	0.03	0.33
BMI	0.07	0.05	1.51	−0.09	0.07	1.66
Stroke	–	–	–	−1.28	0.68	3.54*
Dementia	–	–	–	1.07	0.71	2.23
Cementation	1.10	0.56	3.83*	1.16	0.69	2.83
PH on echo	0.20	1.32	0.02	1.44	1.44	1.01
PH on echo x cementation	−0.83	1.63	0.26	−1.20	1.75	0.47

*Note:* Bolded values = * = *p* < 0.05. BCIS: bone cement impaction syndrome. PH: pulmonary hypertension.

**Table 4:** Multivariate logistic regression results (Preexisting diagnosis of pulmonary hypertension).

*Variable*	BCIS 1			1-year mortality		
	*β*	S.E.	Wald Chi-Square	*β*	S.E.	Wald Chi-Square
Intercept	−5.36	2.96	3.27	1.21	3.26	0.14
Gender	0.37	0.51	0.52	−0.11	0.59	0.03
Age	0.02	0.03	0.66	−0.01	0.03	0.19
BMI	0.08	0.05	2.29	−0.08	0.07	1.14
Stroke	–	–	–	−1.95	0.81	5.84*
Dementia	–	–	–	1.43	0.77	3.46
Cementation	1.45	0.67	4.66*	1.82	0.81	5.06*
Preexisting diagnosis of PH	1.56	1.02	2.34	1.26	1.16	1.17
Preexisting diagnosis of PH x cementation	−1.65	1.21	1.86	−2.65	1.48	3.20

*Note:* Bolded values = * = *p* < 0.05. BCIS: bone cement impaction syndrome. PH: pulmonary hypertension.

#### Pulmonary hypertension on echo.

Pulmonary hypertension on echo was not associated with BCIS I nor 1 year mortality. Cementation significantly predicted BCIS I, such that patients who were cemented were more likely to have BCIS I. Additionally, in the model predicting 1 year mortality, the association between cementation and 1 year mortality was marginally significant in the expected direction; patients who were cemented were more likely to experience 1 year mortality.

#### Moderation.

The interaction term for pulmonary hypertension on echo and cementation did not significantly predict BCIS I nor 1 year mortality.

#### Preexisting diagnosis of pulmonary hypertension.

Preexisting diagnosis of pulmonary hypertension was not associated with BCIS I nor 1 year mortality. Here again, cementation significantly predicted BCIS I and 1 year mortality, such that patients who were cemented were more likely to have BCIS I and 1 year mortality.

#### Moderation.

**Fig 1:**
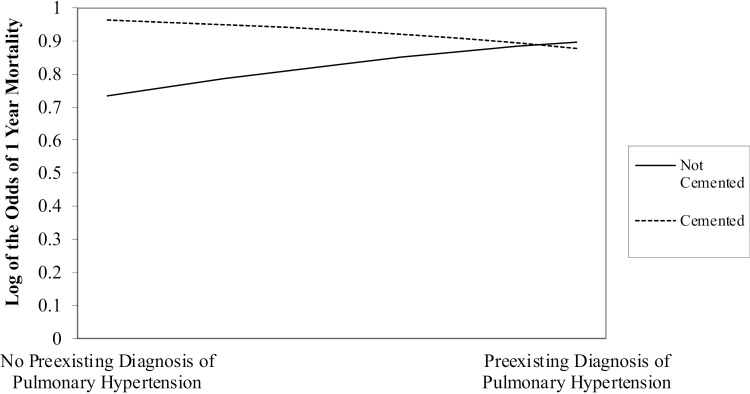
Simple slopes of predicted probabilities of 1-year mortality for patients with and without a pre-existing diagnosis of pulmonary hypertension prior to undergoing hip hemiarthroplasty.

The interaction term for preexisting diagnosis of pulmonary hypertension and cementation did not significantly predict BCIS I. Notably, there was support for the interaction term for preexisting diagnosis of pulmonary hypertension and cementation predicting 1 year mortality; this association was marginal (*p* = .07). Although marginal, the effect was explored further due to the relatively small sample size of the outcome of 1 year mortality (n = 24) and the novelty of the research question. The interaction of preexisting diagnosis of pulmonary hypertension and cementation predicting 1 year mortality was probed to better understand the nature of the interaction. Simple slopes of predicted probabilities are presented in Fig 1 for descriptive purposes. As demonstrated in the figure, there was no effect of cementation on 1 year mortality for patients who had a preexisting diagnosis of pulmonary hypertension. In other words, patients with a preexisting diagnosis of pulmonary hypertension had the same likelihood of 1 year mortality regardless of the type of implant received (cemented versus press-fit). However, among patients who did *not* have a preexisting diagnosis of pulmonary hypertension, the risk of 1 year mortality was significantly higher if they received a cemented implant.

## Discussion

This study did not demonstrate an association between pre-existing diagnosis of PH or echocardiographic criteria of right heart dysfunction and mortality, the study’s primary outcome, after cemented hip hemiarthroplasty. However, cementation was an independent predictor of 1-year mortality and the development of BCIS. Cemented hemiarthroplasty remains the most widely used treatment for displaced geriatric femoral neck fractures (AO/OTA 31-B), as cementation has been associated with modestly superior patient reported outcomes and carries a lower risk of periprosthetic fracture [1,23,24]. Cardiopulmonary morbidity and early perioperative death related to the phenomenon of BCIS have historically been a concern with cemented implants [3–6]. As cementation technique and anesthesia care have improved BCIS is considered largely preventable and of concern only for medically complex patients [2,6–9]. The risks of cementation therefore remains relevant in the setting of geriatric intracapsular hip fractures, where arthroplasty must be urgently undertaken on a non-elective basis in medically frail patients. Patients with PH, regardless of etiology, are considered especially susceptible to BCIS and previous work has suggested that elevated pulmonary arterial pressure, as assessed by TTE, correlates with poor outcomes during cementation undertaken with outdated techniques [4,15]. However, no work to our knowledge, has evaluated if echocardiographic parameters of right heart function correlate with (or are predictive of) patient outcomes following hip hemiarthroplasty performed with modern (fourth generation) cementation techniques.

Although an association between pre-existing diagnosis of PH or echocardiographic criteria of right heart dysfunction and mortality after CH was not found in the current study, previous work which suggested that PH may predispose to BCIS was either theoretical or performed with outdated cementation techniques [4,15]. The theoretical foundation for the association between PH, cementation and cardiopulmonary morbidity has been demonstrated *in vivo* by multiple authors: cemented implant insertion is well-known to result in cardiac emboli formation which have been correlated with temporary pulmonary shunting, decreased stroke volume, and reduced cardiac output [5,25,26]. Donaldson et al. suggested that this embolic load, and the resultant hemodynamic consequences, may be especially poorly tolerated in patients with depressed right heart function at baseline, though they did not cite any data to support this [4]. Leidinger et al. were able to illustrate that 80% (16/20) of patients with elevated pulmonary arterial pressure (>30 mm Hg, as measured on preoperative TTE) developed circulatory insufficiency (defined in this study as requiring vasopressor support with dopamine or ionotropic support with dobutamine greater than 3 μg/kg/min) following cemented stem insertion compared to 18.7% (3/16) with pulmonary artery pressure < 30 mm [15]. However, this relationship was only significant amongst patients who received implants with cement that had not been prepared in a vacuum mixer and did not remain significant in the group of patients who received implants with vacuum-mixed cement. Our results support these authors’ findings, suggesting that echocardiographic findings consistent with right ventricular dysfunction do not correlate well with patient outcomes when modern cementation techniques are used.

Although PH was not associated with mortality in patients who received cemented implants, cementation was an independent predictor of 1-year mortality and the development of BCIS. This further supports previous studies which have illustrated that cementation is an independent risk factor for intraoperative hemodynamic instability and postoperative mortality following hemiarthroplasty [3,11–13,21]. Olsen et al. reported a 21% incidence of BCIS 1 in their retrospective review of 1016 patients who received cemented hemiarthroplasty, identifying [21] ASA 3 & 4 status and COPD diagnosis as other independent risk factors for BCIS [21]. The current study’s 49% incidence of BCIS 1 was notably higher. However, this is perhaps not all that surprising given the inclusion criteria. It is likely that patients who received an echocardiogram within 90 days of their surgery had a higher level of baseline cardiopulmonary comorbidities. Indeed, the average ASA score in the current study was 3.36, versus an average ASA of 2.62 reported by Olsen et al. In a subsequent study comparing 986 patients who received CH to 109 patients who received press fit stems, Olsen et al were able to identify cementation as an independent risk factor for 1-year mortality, with a mortality rate of 13% in the uncemented cohort and a mortality rate of 29% in the cemented cohort. The current study’s results (17% uncemented, 42% cemented) are somewhat higher, though this is likely similarly attributable to the increased medical complexity in this cohort. It should also be noted that all the in-hospital mortalities which occurred in the current study were in patients who received cemented implants. Although these patients were complex with multiple other active medical problems, this finding is not unique as multiple authors have noted that intraoperative and early post-operative deaths (within 48 h of surgery) have been reported almost exclusively in the setting of cemented implant placement [3,11,27].

One of the primary limitations of the current study was the low number of patients who carried a pre-existing diagnosis of pulmonary hypertension. Most of the patients classified as having right heart dysfunction were categorized as such due to echocardiographic findings. Gold standard diagnosis of pulmonary hypertension requires cardiac catheterization and TTE results cannot be used to rule in or out a PH diagnosis [28]. Furthermore, the World Health Organization categorization of the patient’s pulmonary hypertension was not clearly elucidated from chart review, and many patients likely have a combination of contributing factors. Importantly, however, the results of the current project illustrate that the data commonly included in a standard TTE report does not provide information which would help assist with decision-making regarding whether a patient should or should not receive a cemented implant. An additional limitation of the current study in this regard includes the fact that tricuspid annular plane systolic excursion (TAPSE) and tricuspid regurgitation pressure gradient (TRPG) were not routinely collected at our institution during the study period. As a result, these echocardiographic parameters of right heart function were not included in the current analysis and represent an important are of future research [29].

The other primary limitation of the current project includes the fact that the outcomes (mortality, severe BCIS) are relatively low base rate events, even amongst medically frail patients such as those included in this study. Given the low incidence of the outcomes of interest, our results should be viewed as preliminary and interpreted within the context of the wider literature published in this area. Importantly, this study’s findings lend additional support to preliminary findings reported primarily within the European orthopaedic and anesthesia literature, suggesting that cementation may be an independent risk factor for these adverse outcomes [3,11–13,30]. More appropriately powered, prospective, multicenter study is warranted to better understand which patients are at risk for severe adverse outcomes related to cementation.

Finally, this project was also limited by its retrospective design and is at risk of the biases and errors inherent to such projects: The TTE data utilized was collected in a non-standardized fashion by many different clinical staff. The anesthesia chart data was reviewed retrospectively, and it is possible that important changes in patient’s hemodynamic status were not recorded. The selection of stem fixation (cemented versus press fit) was left entirely to surgeon discretion which potentially resulted in selection bias which the current study’s analyses were unable to control for.

In conclusion, the current study supports previous findings, namely that though modern cementation techniques are largely well-tolerated by patients with appropriate cardiopulmonary reserve, certain patients may be predisposed to the development of BCIS. PH, as assessed for by echocardiographic indicators of RV dysfunction, does not appear to be an independent risk factor for the development of BCIS. Medical complexity is a known risk factor for BCIS, though intraoperative and early postoperative mortality related to cementation remain rare. Ultimately, it is important for orthopaedic surgeons to be aware of BCIS and consider press fit fixation as a viable alternative to CH for patients with multiple medical comorbidities.

## Supporting information

S1 File SAS Code. This document contains the SAS code utilized to complete the data analytic strategy.
(DOCX)
