# Supplementary material for: Right heart dysfunction does not increase mortality associated with cemented hemiarthroplasty for hip fractures: A retrospective, preliminary analysis
Source: PLoS One. 2025 Feb 26;20(2):e0318993. doi: 10.1371/journal.pone.0318993 (PMC11864553; doi:10.1371/journal.pone.0318993)
Supplement: S1 File — This document contains the SAS code utilized to complete the data analytic strategy. (DOCX) [file pone.0318993.s001.docx]

*winsorizing to 3 SDs above the mean and log-transforming non-normal data*;

**data** dftwo; set dfone;

if BIPAPCPAPduration > **2.64** then BIPAPCPAPduration = **2.64**;

if lengthICUstay > **8.19** then lengthICUstay = **8.19**;

if Cr > **4.20** then Cr = **4.20**;

hosmortlog = log (hospitalmortality + **1**);

BCIS2log = log (BCIS2 + **1**);

**run**;

*chi square test*;

**PROC** **FREQ** data=df;

TABLES hospitalmortality * cemented / CHISQ;

**RUN**;

****Testing Main effects and Moderation of PH and Cementation on Outcomes*****;

**proc** **logistic** data=df descending;

class subject;

model _1yearmort = gender Ageatsurgery BMI stroke dementia cemented Pulmonaryhypertension cemented*Pulmonaryhypertension;

**run**;

**proc** **logistic** data=df descending;

class subject;

model BCISI = gender Ageatsurgery BMI cemented Pulmonaryhypertension cemented*Pulmonaryhypertension;

**run**;

**proc** **logistic** data=df descending;

class subject;

model _1yearmort = gender Ageatsurgery BMI stroke dementia cemented PulmonaryHTN01 cemented*PulmonaryHTN01;

**run**;

**proc** **logistic** data=df descending;

class subject;

model BCISI = gender Ageatsurgery BMI cemented PulmonaryHTN01 cemented*PulmonaryHTN01;

**run**;
